# Examination of 388 Staphylococcus aureus Isolates from Intensive Care Unit Patients

**DOI:** 10.1128/MRA.01246-19

**Published:** 2019-11-14

**Authors:** Timileyin Adedrian, Stephanie Hitchcock, Lyndsay M. O’Hara, Jane M. Michalski, J. Kristie Johnson, David P. Calfee, Loren G. Miller, Tracy H. Hazen, David A. Rasko, Anthony D. Harris

**Affiliations:** a Institute for Genome Sciences, University of Maryland School of Medicine, Baltimore, Maryland, USA; b Department of Microbiology and Immunology, University of Maryland School of Medicine, Baltimore, Maryland, USA; c Department of Pathology, University of Maryland School of Medicine, Baltimore, Maryland, USA; d Division of Infectious Diseases, Weill Cornell Medicine, New York, New York, USA; e LA Biomedical Research Center at Harbor-UCLA Medical Center, Torrance, California, USA; f Department of Epidemiology and Public Health, University of Maryland School of Medicine, Baltimore, Maryland, USA; Georgia Institute of Technology

## Abstract

We have examined the draft genomes of 388 methicillin-resistant Staphylococcus aureus isolates obtained from intensive care unit patients at three geographically distributed hospitals to determine genomic diversity associated with potential health care worker-associated transmission.

## ANNOUNCEMENT

Antimicrobial resistance has been a constantly evolving problem in the hospital setting (1). This study was undertaken to examine the genomic variation of Staphylococcus aureus isolates obtained from patients in the intensive care unit (ICU). Between January 2016 and August 2018, ICU patients colonized by methicillin-resistant Staphylococcus aureus (MRSA) were enrolled at four hospitals (two in Baltimore, MD, one in Torrance, CA, and one in New York, NY). These patients had a prior surveillance or clinical MRSA culture within 7 days of enrollment. Further details on the patient inclusion criteria and patient demographics are included in a previous publication (2). These isolates were collected as part of a study to examine transmission of Staphylococcus aureus isolates to the gloves and gowns of health care workers during patient contact (2). Each swab was enriched overnight in tryptic soy broth (TSB) with 6.5% salt (Becton Dickinson, Sparks, MD) and plated on CHROMagar Staph aureus medium (Becton Dickinson, Sparks, MD). All rose/mauve colonies were confirmed as S. aureus by Staphaurex latex agglutination and confirmed as MRSA by susceptibility testing following the Clinical and Laboratory Standards Institute guidelines (3). A total of 388 Staphylococcus aureus isolates were collected and examined by whole-genome sequencing.

Genomic DNA was isolated from cultures grown in lysogeny broth overnight. DNA was extracted in 96-well format from 100 μl of sample using the MagAttract PowerMicrobiome DNA/RNA kit (Qiagen, Hilden, Germany), automated on a Hamilton Microlab STAR robotic platform. Bead disruption was conducted on a TissueLyser II (20 Hz for 20 min) instrument in a 96-well deep-well plate in the presence of 200 μl phenol-chloroform. Genomic DNA was eluted in 90 μl water after magnetic bead cleanup. The resulting genomic DNA was quantified by PicoGreen. The sequencing libraries were generated with the Kapa HyperPrep kit (catalog number KK8504) and sequenced on the Illumina HiSeq 4000 using a 2 × 150-bp paired-end kit.

The total number of reads generated for each isolate averaged 3,724,291 bp per genome (Table 1). All software was used with default values. Raw sequencing reads were filtered to remove contaminating phiX reads using BBDuk, one of the BBTools software suite (sourceforge.net/projects/bbmap/). The raw reads were also filtered to remove contaminating Illumina adaptor sequences and quality trimmed using Trimmomatic v. 0.36 (4). The resulting filtered reads were assembled using SPAdes v. 3.13.0 (5). The resulting assemblies were then filtered to contain only contigs longer than 500 bp with a k-mer coverage of ≥5×. Genomes containing more than 500 contigs or an aberrant GC content were removed from further analysis.

The genomes have a mean sequencing coverage of 198× (standard deviation [SD], 58×; minimum [min], 58×; maximum [max], 1,008×). The final assemblies have a mean contig count of 38 (SD, 16; min, 19; max, 199), a mean genome size of 2,857,897 bp (SD, 56,218 bp; min, 2,682,854 bp; max, 3,009,992 bp), a mean GC content of 32.67% (SD, 0.06%; min, 32.56%; max, 33.22%), and a mean *N*_50_ value of 271,123 bp (SD, 89,042 bp; min, 47,630 bp; max, 699,731 bp).

Further analysis will reveal the genetic determinants of transmission via health care worker interactions.

### Data availability.

Relevant statistics, including GenBank and SRA accession numbers for each genome assembly, are included in Table 1.

**TABLE 1:** Genome assembly characteristics

Sample	Insert size (bp)	Total no. of reads	No. of bases sequenced	Genome coverage (×)	No. of contigs	Genome size (bp)	GC content (%)	*N*_50_ (bp)	GenBank accession no.	SRA accession no.
MRSA1	404	2,724,258	411,362,958	147	31	2,791,271	32.62	295,202	WBHV00000000	SRR10179083
MRSA10	493	3,817,952	576,510,752	211	31	2,735,036	32.78	250,902	WBHU00000000	SRR10179082
MRSA100	437	3,630,220	548,163,220	192	35	2,858,562	32.75	170,064	WBHT00000000	SRR10179185
MRSA101	460	3,494,024	527,597,624	186	27	2,838,792	32.62	300,480	WBHS00000000	SRR10179290
MRSA102	420	3,438,980	519,285,980	187	27	2,772,092	32.76	431,335	WBHR00000000	SRR10179017
MRSA103	436	3,348,168	505,573,368	179	31	2,829,018	32.72	443,398	WBHQ00000000	SRR10179006
MRSA104	544	3,590,670	542,191,170	194	29	2,791,200	32.67	699,731	WBHP00000000	SRR10178995
MRSA105	453	3,822,610	577,214,110	200	40	2,887,518	32.65	227,545	WBHO00000000	SRR10178984
MRSA106	545	3,542,228	534,876,428	193	39	2,771,933	32.69	247,058	WBHN00000000	SRR10178973
MRSA107	444	3,791,274	572,482,374	195	28	2,931,006	32.61	284,107	WBHM00000000	SRR10178962
MRSA108	521	5,395,966	814,790,866	284	30	2,873,007	32.73	288,049	WBHL00000000	SRR10178951
MRSA109	516	4,646,288	701,589,488	240	26	2,921,394	32.64	381,525	WBHK00000000	SRR10178940
MRSA11	570	3,440,454	519,508,554	177	29	2,927,157	32.73	287,824	WBHJ00000000	SRR10178929
MRSA110	499	4,174,882	630,407,182	214	36	2,946,853	32.66	283,926	WBHI00000000	SRR10179262
MRSA112	460	3,737,726	564,396,626	195	35	2,887,177	32.65	347,420	WBHH00000000	SRR10179251
MRSA113	490	5,127,932	774,317,732	271	39	2,856,331	32.74	204,938	WBHG00000000	SRR10179240
MRSA114	432	3,427,176	517,503,576	182	34	2,844,520	32.71	243,833	WBHF00000000	SRR10179229
MRSA115	480	2,963,254	447,451,354	153	85	2,924,835	32.70	221,193	WBHE00000000	SRR10179218
MRSA116	464	3,984,944	601,726,544	210	28	2,862,201	32.61	345,460	WBHD00000000	SRR10179207
MRSA117	443	4,100,492	619,174,292	216	32	2,869,996	32.61	333,066	WBHC00000000	SRR10179196
MRSA118	452	3,845,056	580,603,456	202	38	2,877,456	32.64	280,924	WBHB00000000	SRR10179184
MRSA118-1	453	2,658,674	401,459,774	139	39	2,880,266	32.64	198,614	WBHA00000000	SRR10179173
MRSA118-2	465	3,854,178	581,980,878	211	32	2,759,474	32.65	181,622	WBGZ00000000	SRR10179162
MRSA119	542	3,641,384	549,848,984	192	29	2,871,229	32.61	312,042	WBGY00000000	SRR10179151
MRSA12	431	3,292,444	497,159,044	175	38	2,843,273	32.70	242,815	WBGX00000000	SRR10179140
MRSA120	568	3,279,974	495,276,074	174	48	2,851,310	32.73	228,460	WBGW00000000	SRR10179129
MRSA121	530	4,574,424	690,738,024	241	27	2,870,565	32.61	345,387	WBGV00000000	SRR10179118
MRSA122	413	3,287,494	496,411,594	170	58	2,924,929	32.62	158,830	WBGU00000000	SRR10179107
MRSA123	399	2,911,336	439,611,736	158	43	2,783,732	32.69	154,081	WBGT00000000	SRR10179312
MRSA124	445	3,611,020	545,264,020	187	27	2,915,523	32.66	333,124	WBGS00000000	SRR10179301
MRSA125	463	3,311,634	500,056,734	174	34	2,869,237	32.61	304,481	WBGR00000000	SRR10179289
MRSA126	473	3,640,022	549,643,322	190	38	2,897,949	32.56	235,657	WBGQ00000000	SRR10179278
MRSA127	450	3,526,420	532,489,420	183	33	2,912,704	32.65	304,669	WBGP00000000	SRR10179095
MRSA128	502	3,523,528	532,052,728	183	30	2,909,559	32.64	333,118	WBGO00000000	SRR10179084
MRSA129	438	3,439,930	519,429,430	188	32	2,763,701	32.72	226,266	WBGN00000000	SRR10179071
MRSA13	402	5,828,122	880,046,422	301	32	2,925,776	32.62	158,970	WBGM00000000	SRR10179060
MRSA130	447	3,153,906	476,239,806	164	32	2,897,923	32.64	284,122	WBGL00000000	SRR10179049
MRSA131	457	3,798,068	573,508,268	209	37	2,749,664	32.66	153,446	WBGK00000000	SRR10179038
MRSA132	462	4,012,102	605,827,402	218	34	2,776,634	32.73	270,629	WBGJ00000000	SRR10179027
MRSA133	417	3,300,814	498,422,914	184	25	2,713,650	32.69	238,316	WBGI00000000	SRR10179018
MRSA134	446	3,068,042	463,274,342	161	28	2,881,857	32.66	226,269	WBGH00000000	SRR10179016
MRSA135	496	3,128,940	472,469,940	162	31	2,907,575	32.64	304,501	WBGG00000000	SRR10179015
MRSA136	471	3,625,842	547,502,142	188	27	2,918,501	32.64	381,526	WBGF00000000	SRR10179014
MRSA137	427	3,121,708	471,377,908	164	37	2,866,864	32.68	293,060	WBGE00000000	SRR10179013
MRSA138	501	3,539,406	534,450,306	183	30	2,917,399	32.64	346,822	WBGD00000000	SRR10179012
MRSA139	454	3,407,166	514,482,066	183	39	2,807,994	32.71	174,640	WBGC00000000	SRR10179011
MRSA14	534	4,257,130	642,826,630	222	33	2,889,505	32.71	290,683	WBGB00000000	SRR10179010
MRSA140	483	3,802,934	574,243,034	203	42	2,833,831	32.71	243,368	WBGA00000000	SRR10179009
MRSA141	535	3,725,206	562,506,106	196	22	2,870,417	32.66	373,724	WBFZ00000000	SRR10179008
MRSA142	419	2,092,822	316,016,122	112	32	2,821,431	32.61	265,134	WBFY00000000	SRR10179007
MRSA143	480	4,343,128	655,812,328	230	29	2,846,091	32.74	221,191	WBFX00000000	SRR10179005
MRSA144	444	3,991,868	602,772,068	213	34	2,826,597	32.78	441,860	WBFW00000000	SRR10179004
MRSA145	468	3,009,380	454,416,380	156	49	2,906,043	32.74	211,906	WBFV00000000	SRR10179003
MRSA146	425	3,626,574	547,612,674	197	31	2,773,222	32.71	311,318	WBFU00000000	SRR10179002
MRSA147	433	3,460,602	522,550,902	184	36	2,846,705	32.73	222,180	WBFT00000000	SRR10179001
MRSA149	448	3,748,290	565,991,790	197	47	2,868,668	32.70	175,740	WBFS00000000	SRR10179000
MRSA15	546	3,996,568	603,481,768	207	91	2,918,071	32.71	80,371	WBFR00000000	SRR10178999
MRSA150	508	4,574,438	690,740,138	241	28	2,862,988	32.61	345,454	WBFQ00000000	SRR10178998
MRSA151	629	4,660,432	703,725,232	241	34	2,918,538	32.64	213,199	WBFP00000000	SRR10178997
MRSA152	408	2,937,352	443,540,152	156	35	2,848,038	32.76	428,845	WBFO00000000	SRR10178996
MRSA153	445	3,008,712	454,315,512	162	25	2,795,883	32.68	329,230	WBFN00000000	SRR10178994
MRSA154	447	4,296,358	648,750,058	223	26	2,908,809	32.64	345,506	WBFM00000000	SRR10178993
MRSA155	463	3,979,074	600,840,174	215	27	2,792,725	32.76	443,401	WBFL00000000	SRR10178992
MRSA156	516	4,463,524	673,992,124	231	28	2,911,879	32.65	346,821	WBFK00000000	SRR10178991
MRSA157	486	4,162,340	628,513,340	218	38	2,882,254	32.61	283,671	WBFJ00000000	SRR10178990
MRSA158	466	2,926,016	441,828,416	154	33	2,869,350	32.61	333,123	WBFI00000000	SRR10178989
MRSA159	565	3,870,056	584,378,456	204	25	2,867,342	32.61	381,216	WBFH00000000	SRR10178988
MRSA16	494	4,106,074	620,017,174	212	30	2,920,198	32.64	345,464	WBFG00000000	SRR10178987
MRSA160	471	3,746,130	565,665,630	194	35	2,912,459	32.64	283,804	WBFF00000000	SRR10178986
MRSA161	457	2,973,608	449,014,808	158	32	2,833,955	32.76	469,494	WBFE00000000	SRR10178985
MRSA162	426	2,544,208	384,175,408	140	35	2,743,758	32.75	222,187	WBFD00000000	SRR10178983
MRSA163	367	3,132,986	473,080,886	168	30	2,824,194	32.78	419,318	WBFC00000000	SRR10178982
MRSA164	426	2,670,332	403,220,132	140	21	2,887,655	32.68	405,200	WBFB00000000	SRR10178981
MRSA165	422	3,264,376	492,920,776	174	53	2,827,763	32.78	209,148	WBFA00000000	SRR10178980
MRSA166	556	4,212,704	636,118,304	226	36	2,811,530	32.69	177,209	WBEZ00000000	SRR10178979
MRSA167	434	3,461,352	522,664,152	182	50	2,866,485	32.71	212,302	WBEY00000000	SRR10178978
MRSA168	425	3,613,478	545,635,178	191	34	2,861,274	32.75	243,955	WBEX00000000	SRR10178977
MRSA169	418	3,838,020	579,541,020	205	35	2,831,356	32.74	221,030	WBEW00000000	SRR10178976
MRSA17	523	4,144,954	625,888,054	221	42	2,826,838	32.75	288,279	WBEV00000000	SRR10178975
MRSA170	525	3,805,896	574,690,296	202	25	2,841,075	32.63	333,123	WBEU00000000	SRR10178974
MRSA171	507	4,015,922	606,404,222	210	30	2,893,518	32.65	333,118	WBET00000000	SRR10178972
MRSA172	501	3,860,830	582,985,330	200	25	2,911,523	32.63	333,119	WBES00000000	SRR10178971
MRSA173	443	2,972,436	448,837,836	167	40	2,682,854	32.69	127,559	WBER00000000	SRR10178970
MRSA174	468	3,387,432	511,502,232	175	33	2,914,617	32.64	333,125	WBEQ00000000	SRR10178969
MRSA175	482	3,604,394	544,263,494	190	26	2,867,625	32.61	345,459	WBEP00000000	SRR10178968
MRSA176	514	2,837,280	428,429,280	147	35	2,913,562	32.63	243,791	WBEO00000000	SRR10178967
MRSA177	469	3,547,212	535,629,012	189	27	2,839,451	32.62	333,117	WBEN00000000	SRR10178966
MRSA178	425	2,377,292	358,971,092	127	30	2,835,593	32.63	284,012	WBEM00000000	SRR10178965
MRSA179	409	2,698,544	407,480,144	144	29	2,828,486	32.62	280,525	WBEL00000000	SRR10178964
MRSA18	552	4,039,484	609,962,084	220	24	2,767,335	32.69	432,938	WBEK00000000	SRR10178963
MRSA180-2	460	4,057,392	612,666,192	213	25	2,879,538	32.67	348,739	WBEJ00000000	SRR10178961
MRSA181	416	3,129,838	472,605,538	162	28	2,916,323	32.64	304,504	WBEI00000000	SRR10178960
MRSA182	470	3,764,934	568,505,034	202	33	2,818,901	32.69	243,942	WBEH00000000	SRR10178959
MRSA183	468	3,085,020	465,838,020	165	30	2,818,259	32.61	381,214	WBEG00000000	SRR10178958
MRSA184	450	4,281,728	646,540,928	229	43	2,824,325	32.62	284,137	WBEF00000000	SRR10178957
MRSA185	446	3,094,626	467,288,526	164	30	2,852,201	32.73	260,992	WBEE00000000	SRR10178956
MRSA188	408	3,945,442	595,761,742	211	33	2,819,824	32.77	419,310	WBED00000000	SRR10178955
MRSA189	424	2,792,780	421,709,780	148	32	2,842,082	32.74	240,598	WBEC00000000	SRR10178954
MRSA19	432	2,755,104	416,020,704	142	41	2,936,717	32.69	296,927	WBEB00000000	SRR10178953
MRSA190	467	3,319,368	501,224,568	173	33	2,901,213	32.56	304,667	WBEA00000000	SRR10178952
MRSA191	475	3,838,368	579,593,568	214	33	2,704,186	32.65	181,767	WBDZ00000000	SRR10178950
MRSA192	455	2,968,666	448,268,566	158	47	2,829,462	32.76	443,400	WBDY00000000	SRR10178949
MRSA193	431	2,574,638	388,770,338	133	62	2,926,664	32.69	172,779	WBDX00000000	SRR10178948
MRSA194	449	3,670,634	554,265,734	197	34	2,816,505	32.62	282,854	WBDW00000000	SRR10178947
MRSA195	419	6,258,220	944,991,220	330	45	2,867,339	32.77	221,192	WBDV00000000	SRR10178946
MRSA196	450	3,368,864	508,698,464	176	68	2,897,982	32.72	74,602	WBDU00000000	SRR10178945
MRSA197	434	3,269,268	493,659,468	173	38	2,849,256	32.75	221,191	WBDT00000000	SRR10178944
MRSA198	437	3,447,082	520,509,382	182	48	2,861,081	32.76	240,041	WBDS00000000	SRR10178943
MRSA199	431	2,727,950	411,920,450	150	33	2,753,324	32.76	187,458	WBDR00000000	SRR10178942
MRSA2	413	3,220,458	486,289,158	171	46	2,850,144	32.74	137,200	WBDQ00000000	SRR10178941
MRSA20	457	3,731,134	563,401,234	194	30	2,911,511	32.64	304,377	WBDP00000000	SRR10178939
MRSA200	484	3,484,356	526,137,756	182	38	2,896,754	32.64	291,060	WBDO00000000	SRR10178938
MRSA201	431	3,048,166	460,273,066	164	37	2,811,146	32.70	158,127	WBDN00000000	SRR10178937
MRSA202	438	346,096	52,260,496	18	78	2,879,624	32.65	76,633	WBDM00000000	SRR10178936
MRSA203	494	3,249,902	490,735,202	169	27	2,910,008	32.63	345,459	WBDL00000000	SRR10178935
MRSA204	470	3,669,440	554,085,440	191	41	2,895,554	32.69	288,336	WBDK00000000	SRR10178934
MRSA205	429	3,566,724	538,575,324	188	30	2,870,246	32.61	345,440	WBDJ00000000	SRR10178933
MRSA206	536	3,813,618	575,856,318	202	42	2,847,155	32.73	244,036	WBDI00000000	SRR10178932
MRSA207	416	3,292,136	497,112,536	172	33	2,884,574	32.70	205,001	WBDH00000000	SRR10178931
MRSA209	507	3,661,926	552,950,826	188	45	2,935,815	32.71	221,189	WBDG00000000	SRR10178930
MRSA21	515	4,170,864	629,800,464	212	87	2,966,182	32.63	74,602	WBDF00000000	SRR10178928
MRSA211	432	6,579,076	993,440,476	341	30	2,915,317	32.63	304,502	WBDE00000000	SRR10179271
MRSA212	483	3,744,984	565,492,584	199	29	2,835,036	32.63	345,459	WBDD00000000	SRR10179270
MRSA213	501	3,639,624	549,583,224	200	33	2,749,708	32.64	238,202	WBDC00000000	SRR10179269
MRSA214	530	3,942,756	595,356,156	208	26	2,866,675	32.61	283,744	WBDB00000000	SRR10179268
MRSA215	492	3,731,276	563,422,676	197	43	2,866,115	32.76	221,190	WBDA00000000	SRR10179267
MRSA217	459	4,298,220	649,031,220	227	31	2,857,735	32.64	305,071	WBCZ00000000	SRR10179266
MRSA218	465	4,117,258	621,705,958	211	37	2,946,256	32.66	345,390	WBCY00000000	SRR10179265
MRSA219	418	4,099,586	619,037,486	216	45	2,865,418	32.76	153,754	WBCX00000000	SRR10179264
MRSA22	446	3,261,576	492,497,976	171	47	2,871,732	32.76	419,332	WBCW00000000	SRR10179263
MRSA220	488	4,659,914	703,647,014	246	25	2,864,498	32.60	345,476	WBCV00000000	SRR10179261
MRSA221	457	2,672,266	403,512,166	149	27	2,706,207	32.72	238,315	WBCU00000000	SRR10179260
MRSA222	512	3,806,634	574,801,734	199	28	2,881,882	32.65	379,481	WBCT00000000	SRR10179259
MRSA223	409	3,220,758	486,334,458	167	25	2,905,583	32.64	344,326	WBCS00000000	SRR10179258
MRSA224	509	4,842,994	731,292,094	256	37	2,859,654	32.75	170,064	WBCR00000000	SRR10179257
MRSA225	436	3,257,784	491,925,384	176	27	2,794,371	32.62	375,038	WBCQ00000000	SRR10179256
MRSA226	565	4,013,544	606,045,144	211	26	2,869,844	32.61	333,055	WBCP00000000	SRR10179255
MRSA227	469	3,827,720	577,985,720	200	42	2,884,205	32.57	190,424	WBCO00000000	SRR10179254
MRSA228	572	3,851,790	581,620,290	201	27	2,891,072	32.66	375,324	WBCN00000000	SRR10179253
MRSA229	420	3,056,396	461,515,796	159	42	2,902,392	32.56	284,051	WBCM00000000	SRR10179252
MRSA23	471	2,997,380	452,604,380	159	38	2,841,517	32.74	244,037	WBCL00000000	SRR10179250
MRSA230	523	3,311,574	500,047,674	174	42	2,870,984	32.76	218,466	WBCK00000000	SRR10179249
MRSA231	549	3,666,928	553,706,128	193	27	2,866,599	32.63	299,350	WBCJ00000000	SRR10179248
MRSA232	471	3,234,636	488,430,036	173	28	2,819,922	32.60	287,014	WBCI00000000	SRR10179247
MRSA233	532	3,565,798	538,435,498	191	36	2,812,542	32.69	244,439	WBCH00000000	SRR10179246
MRSA234	512	3,720,848	561,848,048	196	29	2,868,283	32.61	304,667	WBCG00000000	SRR10179245
MRSA235	457	3,313,776	500,380,176	179	30	2,802,757	32.69	243,947	WBCF00000000	SRR10179244
MRSA236	458	3,815,344	576,116,944	197	27	2,917,856	32.64	381,528	WBCE00000000	SRR10179243
MRSA237	481	4,063,250	613,550,750	213	32	2,875,033	32.59	345,465	WBCD00000000	SRR10179242
MRSA238	427	6,968,666	1,052,268,566	363	24	2,901,533	32.64	345,509	WBCC00000000	SRR10179241
MRSA239	450	3,391,526	512,120,426	174	43	2,942,961	32.58	345,454	WBCB00000000	SRR10179239
MRSA240	563	3,694,530	557,874,030	196	49	2,844,090	32.64	207,199	WBCA00000000	SRR10179238
MRSA241	431	2,834,082	427,946,382	156	30	2,751,102	32.70	226,145	WBBZ00000000	SRR10179237
MRSA243	381	2,963,456	447,481,856	162	47	2,760,329	32.69	173,135	WBBY00000000	SRR10179236
MRSA244	461	4,082,526	616,461,426	215	26	2,865,586	32.60	345,460	WBBX00000000	SRR10179235
MRSA245	516	4,774,468	720,944,668	250	50	2,879,787	32.73	218,010	WBBW00000000	SRR10179234
MRSA246	473	4,178,766	630,993,666	217	31	2,911,770	32.63	345,460	WBBV00000000	SRR10179233
MRSA247	440	2,929,758	442,393,458	157	56	2,810,088	32.72	161,964	WBBU00000000	SRR10179232
MRSA248	462	3,070,682	463,672,982	166	29	2,801,180	32.63	220,511	WBBT00000000	SRR10179231
MRSA249	527	3,320,236	501,355,636	175	44	2,862,450	32.77	218,466	WBBS00000000	SRR10179230
MRSA25	421	3,155,710	476,512,210	169	32	2,826,454	32.73	244,212	WBBR00000000	SRR10179228
MRSA250	571	4,938,280	745,680,280	256	26	2,912,501	32.64	375,333	WBBQ00000000	SRR10179227
MRSA251	470	4,001,954	604,295,054	219	30	2,765,472	32.78	173,797	WBBP00000000	SRR10179226
MRSA252	509	4,187,304	632,282,904	222	34	2,841,760	32.62	191,135	WBBO00000000	SRR10179225
MRSA253	489	4,323,678	652,875,378	228	44	2,867,637	32.76	221,190	WBBN00000000	SRR10179224
MRSA254	492	4,927,710	744,084,210	253	34	2,936,624	32.66	329,326	WBBM00000000	SRR10179223
MRSA255	412	3,133,588	473,171,788	165	36	2,861,196	32.61	280,662	WBBL00000000	SRR10179222
MRSA256	492	5,068,778	765,385,478	268	58	2,851,223	32.59	284,068	WBBK00000000	SRR10179221
MRSA257	500	3,637,532	549,267,332	199	28	2,766,681	32.67	243,907	WBBJ00000000	SRR10179220
MRSA258	445	4,335,288	654,628,488	235	39	2,789,678	32.75	205,203	WBBI00000000	SRR10179219
MRSA259	542	3,541,892	534,825,692	182	42	2,936,350	32.69	284,130	WBBH00000000	SRR10179217
MRSA26	441	2,945,322	444,743,622	156	26	2,852,277	32.73	237,629	WBBG00000000	SRR10179216
MRSA260	491	4,821,822	728,095,122	255	31	2,856,777	32.71	222,178	WBBF00000000	SRR10179215
MRSA261	498	4,207,052	635,264,852	233	21	2,721,604	32.69	495,750	WBBE00000000	SRR10179214
MRSA262	503	3,573,532	539,603,332	190	37	2,842,570	32.75	216,081	WBBD00000000	SRR10179213
MRSA263	535	3,871,206	584,552,106	200	32	2,918,245	32.63	345,466	WBBC00000000	SRR10179212
MRSA264	536	3,627,814	547,799,914	187	46	2,935,729	32.70	241,076	WBBB00000000	SRR10179211
MRSA265	474	3,283,426	495,797,326	177	31	2,793,647	32.71	218,367	WBBA00000000	SRR10179210
MRSA266	421	5,861,928	885,151,128	298	46	2,967,435	32.61	283,813	WBAZ00000000	SRR10179209
MRSA267	472	3,859,058	582,717,758	200	30	2,911,659	32.65	304,668	WBAY00000000	SRR10179208
MRSA268	425	3,354,030	506,458,530	174	85	2,914,347	32.61	109,368	WBAX00000000	SRR10179206
MRSA269	495	3,923,022	592,376,322	207	57	2,867,422	32.73	154,109	WBAW00000000	SRR10179205
MRSA27	489	4,335,490	654,658,990	239	28	2,742,726	32.73	222,215	WBAV00000000	SRR10179204
MRSA271	431	3,037,012	458,588,812	158	35	2,896,468	32.66	201,609	WBAU00000000	SRR10179203
MRSA272	447	3,784,542	571,465,842	195	114	2,928,368	32.63	71,498	WBAT00000000	SRR10179202
MRSA273	434	3,413,422	515,426,722	183	38	2,816,195	32.65	267,012	WBAS00000000	SRR10179201
MRSA274	455	3,866,870	583,897,370	206	37	2,839,459	32.75	331,131	WBAR00000000	SRR10179200
MRSA275	449	3,634,216	548,766,616	196	29	2,805,965	32.71	240,324	WBAQ00000000	SRR10179199
MRSA276	486	3,885,360	586,689,360	214	32	2,740,467	32.67	171,162	WBAP00000000	SRR10179198
MRSA277	448	3,351,676	506,103,076	176	28	2,870,423	32.61	345,357	WBAO00000000	SRR10179197
MRSA278	450	4,033,918	609,121,618	212	35	2,868,455	32.63	271,788	WBAN00000000	SRR10179195
MRSA279	490	4,018,472	606,789,272	212	24	2,866,241	32.61	345,481	WBAM00000000	SRR10179194
MRSA28	501	4,872,908	735,809,108	251	37	2,927,715	32.72	221,191	WBAL00000000	SRR10179193
MRSA280	456	3,819,212	576,701,012	203	26	2,846,942	32.71	419,197	WBAK00000000	SRR10179192
MRSA281	457	3,118,280	470,860,280	164	33	2,875,702	32.64	235,774	WBAJ00000000	SRR10179191
MRSA282	437	3,198,696	483,003,096	166	48	2,913,353	32.69	418,353	WBAI00000000	SRR10179190
MRSA283	417	1,945,088	293,708,288	104	35	2,814,763	32.80	222,055	WBAH00000000	SRR10179189
MRSA285	441	4,000,778	604,117,478	220	32	2,741,201	32.67	176,733	WBAF00000000	SRR10179187
MRSA286	464	4,089,866	617,569,766	215	35	2,869,967	32.60	383,639	WBAE00000000	SRR10179186
MRSA288	440	3,430,916	518,068,316	179	32	2,897,102	32.65	215,804	WBAD00000000	SRR10179183
MRSA289	430	5,884,810	888,606,310	305	28	2,915,143	32.64	381,528	WBAC00000000	SRR10179182
MRSA29	452	4,356,220	657,789,220	226	30	2,912,471	32.65	304,553	WBAB00000000	SRR10179181
MRSA290	479	3,476,088	524,889,288	180	31	2,923,075	32.62	345,710	WBAA00000000	SRR10179180
MRSA291	462	3,549,742	536,011,042	185	44	2,903,180	32.79	221,197	WAZZ00000000	SRR10179179
MRSA292	439	6,322,490	954,695,990	340	26	2,806,629	32.60	304,688	WAZY00000000	SRR10179178
MRSA293	460	3,476,848	525,004,048	180	51	2,919,171	32.74	188,162	WAZX00000000	SRR10179177
MRSA294	459	3,669,348	554,071,548	199	29	2,783,007	32.62	282,791	WAZW00000000	SRR10179176
MRSA295	478	3,187,154	481,260,254	165	30	2,908,039	32.62	333,119	WAZV00000000	SRR10179175
MRSA297	439	3,000,452	453,068,252	162	38	2,793,766	32.67	185,500	WAZU00000000	SRR10179174
MRSA297_2	436	6,095,952	920,488,752	315	32	2,919,616	32.64	345,439	WAZT00000000	SRR10179172
MRSA298	439	871,450	131,588,950	46	56	2,867,339	32.63	109,627	WAZS00000000	SRR10179171
MRSA299	454	2,991,914	451,779,014	157	66	2,874,125	32.68	79,250	WAZR00000000	SRR10179170
MRSA3	498	3,779,774	570,745,874	209	38	2,733,468	32.69	235,136	WAZQ00000000	SRR10179169
MRSA30	419	3,343,824	504,917,424	173	34	2,924,667	32.64	382,340	WAZP00000000	SRR10179168
MRSA300	412	2,791,040	421,447,040	144	60	2,920,255	33.22	215,700	WAZO00000000	SRR10179167
MRSA301	446	2,895,826	437,269,726	150	40	2,920,164	32.67	252,755	WAZN00000000	SRR10179166
MRSA302	520	3,018,578	455,805,278	159	30	2,863,042	32.62	304,666	WAZM00000000	SRR10179165
MRSA303	449	2,978,026	449,681,926	166	45	2,702,362	32.68	108,857	WAZL00000000	SRR10179164
MRSA304	462	3,316,166	500,741,066	176	37	2,848,927	32.63	283,747	WAZK00000000	SRR10179163
MRSA305	484	3,283,344	495,784,944	172	48	2,890,519	32.76	188,508	WAZJ00000000	SRR10179161
MRSA306	451	2,400,340	362,451,340	124	37	2,915,564	32.66	284,020	WAZI00000000	SRR10179160
MRSA307	435	5,882,130	888,201,630	305	37	2,913,784	32.64	287,311	WAZH00000000	SRR10179159
MRSA309	399	2,382,860	359,811,860	128	40	2,819,904	32.80	222,096	WAZG00000000	SRR10179158
MRSA31	504	4,058,140	612,779,140	211	35	2,901,655	32.66	382,058	WAZF00000000	SRR10179157
MRSA33	485	4,191,826	632,965,726	225	27	2,808,307	32.61	304,754	WAZE00000000	SRR10179156
MRSA34	503	4,335,280	654,627,280	230	33	2,847,795	32.71	250,196	WAZD00000000	SRR10179155
MRSA35	460	4,467,540	674,598,540	238	48	2,835,753	32.73	201,417	WAZC00000000	SRR10179154
MRSA36	477	4,217,736	636,878,136	222	49	2,875,106	32.76	221,190	WAZB00000000	SRR10179153
MRSA37	449	3,725,368	562,530,568	202	35	2,790,040	32.69	173,933	WAZA00000000	SRR10179152
MRSA38	493	3,615,778	545,982,478	199	26	2,738,669	32.74	244,213	WAYZ00000000	SRR10179150
MRSA4	458	4,157,464	627,777,064	219	70	2,869,449	32.69	144,005	WAYY00000000	SRR10179149
MRSA40	469	3,773,764	569,838,364	197	60	2,893,606	32.75	221,191	WAYX00000000	SRR10179148
MRSA41	511	3,545,850	535,423,350	187	29	2,864,890	32.61	333,124	WAYW00000000	SRR10179147
MRSA42	414	2,747,998	414,947,698	141	49	2,940,783	32.60	173,124	WAYV00000000	SRR10179146
MRSA43	443	3,221,830	486,496,330	169	63	2,881,679	32.76	221,197	WAYU00000000	SRR10179145
MRSA44	559	5,006,862	756,036,162	257	47	2,940,061	32.69	297,379	WAYT00000000	SRR10179144
MRSA45	458	5,689,392	859,098,192	303	34	2,839,319	32.64	299,492	WAYS00000000	SRR10179143
MRSA46	500	4,137,060	624,696,060	214	47	2,914,102	32.74	234,562	WAYR00000000	SRR10179142
MRSA47	552	3,574,842	539,801,142	188	45	2,872,312	32.68	153,241	WAYQ00000000	SRR10179141
MRSA48	425	2,379,424	359,293,024	127	29	2,830,794	32.68	300,350	WAYP00000000	SRR10179139
MRSA5	483	4,601,064	694,760,664	240	35	2,894,503	32.79	288,710	WAYO00000000	SRR10179138
MRSA50	451	3,390,568	511,975,768	177	110	2,896,131	32.64	67,684	WAYN00000000	SRR10179137
MRSA500	435	3,415,458	515,734,158	179	30	2,880,874	32.72	443,401	WAWM00000000	SRR10179295
MRSA501	443	4,298,628	649,092,828	226	28	2,873,289	32.60	377,950	WAWL00000000	SRR10179294
MRSA502	470	4,036,966	609,581,866	221	19	2,752,110	32.71	319,610	WAWK00000000	SRR10179293
MRSA503	436	2,955,596	446,294,996	157	30	2,847,936	32.71	243,826	WAWJ00000000	SRR10179292
MRSA504	427	3,025,994	456,925,094	160	26	2,851,822	32.61	381,190	WAWI00000000	SRR10179291
MRSA505	440	2,601,462	392,820,762	141	41	2,783,503	32.70	174,478	WAWH00000000	SRR10179288
MRSA506	467	3,289,676	496,741,076	173	44	2,863,665	32.75	419,246	WAWG00000000	SRR10179287
MRSA508	427	3,276,268	494,716,468	174	63	2,847,855	32.66	180,953	WAWF00000000	SRR10179286
MRSA509	470	3,231,040	487,887,040	164	77	2,967,220	32.67	182,962	WAWE00000000	SRR10179285
MRSA51	522	3,697,536	558,327,936	192	44	2,911,059	32.67	221,191	WAYM00000000	SRR10179136
MRSA510	465	3,865,744	583,727,344	205	26	2,844,843	32.71	316,224	WAWD00000000	SRR10179284
MRSA511	479	3,489,826	526,963,726	183	29	2,878,887	32.63	333,150	WAWC00000000	SRR10179283
MRSA512	472	3,175,052	479,432,852	165	34	2,899,500	32.63	283,511	WAWB00000000	SRR10179282
MRSA513	476	4,407,928	665,597,128	230	38	2,889,548	32.75	243,955	WAWA00000000	SRR10179281
MRSA514	519	4,551,784	687,319,384	241	42	2,850,571	32.74	295,426	WAVZ00000000	SRR10179280
MRSA515	416	3,262,576	492,648,976	171	67	2,879,312	32.67	95,743	WAVY00000000	SRR10179279
MRSA516	469	3,242,860	489,671,860	171	33	2,859,930	32.74	229,079	WAVX00000000	SRR10179277
MRSA517	458	2,666,338	402,617,038	147	31	2,739,909	32.64	193,778	WAVW00000000	SRR10179276
MRSA518	459	3,482,392	525,841,192	188	38	2,798,003	32.71	150,800	WAVV00000000	SRR10179275
MRSA52	518	4,825,326	728,624,226	255	30	2,857,882	32.64	333,047	WAYL00000000	SRR10179135
MRSA520	494	3,931,276	593,622,676	211	47	2,816,342	32.61	187,117	WAVU00000000	SRR10179274
MRSA521	549	3,197,898	482,882,598	166	70	2,903,287	32.63	90,077	WAVT00000000	SRR10179273
MRSA522	489	3,514,086	530,626,986	189	30	2,804,992	32.76	244,193	WAVS00000000	SRR10179272
MRSA523	429	3,026,854	457,054,954	157	59	2,902,336	32.65	184,311	WAVR00000000	SRR10179099
MRSA524	478	3,462,396	522,821,796	186	57	2,814,381	32.62	180,937	WAVQ00000000	SRR10179098
MRSA525	459	4,174,068	630,284,268	218	53	2,888,019	32.74	419,381	WAVP00000000	SRR10179097
MRSA526	484	4,116,900	621,651,900	226	39	2,756,328	32.63	206,464	WAVO00000000	SRR10179096
MRSA527	456	3,848,958	581,192,658	202	30	2,880,371	32.58	284,170	WAVN00000000	SRR10179094
MRSA528	463	2,815,264	425,104,864	149	32	2,844,949	32.72	221,188	WAVM00000000	SRR10179093
MRSA529	492	3,102,740	468,513,740	161	29	2,914,637	32.63	283,689	WAVL00000000	SRR10179092
MRSA53	493	4,372,378	660,229,078	227	79	2,904,045	32.76	221,191	WAYK00000000	SRR10179134
MRSA530	465	3,052,098	460,866,798	168	43	2,740,166	32.66	120,736	WAVK00000000	SRR10179091
MRSA531	466	3,341,662	504,590,962	183	27	2,760,669	32.65	187,382	WAVJ00000000	SRR10179090
MRSA532	507	3,214,462	485,383,762	171	39	2,833,992	32.69	283,942	WAVI00000000	SRR10179089
MRSA533	557	5,072,588	765,960,788	273	30	2,804,840	32.69	185,354	WAVH00000000	SRR10179088
MRSA534	494	4,158,004	627,858,604	217	39	2,890,472	32.74	243,944	WAVG00000000	SRR10179087
MRSA535	482	3,087,632	466,232,432	160	33	2,912,001	32.64	223,450	WAVF00000000	SRR10179086
MRSA536	484	3,604,420	544,267,420	189	43	2,880,990	32.73	243,942	WAVE00000000	SRR10179085
MRSA537	470	4,508,726	680,817,626	240	40	2,842,293	32.77	212,343	WAVD00000000	SRR10179081
MRSA538	471	3,667,442	553,783,742	197	24	2,817,554	32.63	345,445	WAVC00000000	SRR10179080
MRSA539	498	3,261,246	492,448,146	172	27	2,868,566	32.61	588,580	WAVB00000000	SRR10179079
MRSA54	523	4,084,308	616,730,508	221	41	2,792,637	32.66	222,167	WAYJ00000000	SRR10179133
MRSA540	542	1,209,052	182,566,852	64	37	2,846,408	32.73	222,067	WAVA00000000	SRR10179078
MRSA541	489	2,325,176	351,101,576	122	27	2,869,770	32.60	382,034	WAUZ00000000	SRR10179077
MRSA542	462	2,780,540	419,861,540	152	27	2,763,808	32.68	244,037	WAUY00000000	SRR10179076
MRSA543	473	2,680,578	404,767,278	140	74	2,892,297	32.70	221,191	WAUX00000000	SRR10179075
MRSA55	518	4,010,370	605,565,870	211	31	2,865,617	32.63	304,484	WAYI00000000	SRR10179132
MRSA56	492	3,953,948	597,046,148	203	25	2,944,945	32.60	405,519	WAYH00000000	SRR10179131
MRSA57	471	3,381,960	510,675,960	175	76	2,915,774	32.66	76,967	WAYG00000000	SRR10179130
MRSA58	531	4,692,672	708,593,472	240	38	2,946,630	32.66	304,668	WAYF00000000	SRR10179128
MRSA59	476	3,644,390	550,302,890	196	24	2,805,747	32.71	243,818	WAYE00000000	SRR10179127
MRSA6	501	3,890,944	587,532,544	205	36	2,870,931	32.62	208,319	WAYD00000000	SRR10179126
MRSA60	487	5,306,022	801,209,322	275	34	2,913,198	32.64	283,748	WAYC00000000	SRR10179125
MRSA61	446	3,275,728	494,634,928	172	27	2,869,015	32.61	381,210	WAYB00000000	SRR10179124
MRSA62	518	5,131,476	774,852,876	266	35	2,912,904	32.63	333,118	WAYA00000000	SRR10179123
MRSA63	563	3,776,710	570,283,210	189	145	3,009,992	32.58	66,240	WAXZ00000000	SRR10179122
MRSA64	484	3,823,966	577,418,866	198	29	2,913,750	32.63	331,613	WAXY00000000	SRR10179121
MRSA65	465	4,051,000	611,701,000	218	30	2,804,978	32.61	283,749	WAXX00000000	SRR10179120
MRSA66-1	474	4,445,738	671,306,438	240	29	2,800,438	32.63	329,168	WAXW00000000	SRR10179119
MRSA66-2	469	3,410,614	515,002,714	178	32	2,892,495	32.66	304,500	WAXV00000000	SRR10179117
MRSA67	533	4,128,542	623,409,842	224	50	2,779,612	32.69	114,673	WAXU00000000	SRR10179116
MRSA68	512	4,838,382	730,595,682	256	28	2,854,912	32.64	286,833	WAXT00000000	SRR10179115
MRSA69	496	4,624,496	698,298,896	241	25	2,900,596	32.65	602,546	WAXS00000000	SRR10179114
MRSA7	559	3,281,582	495,518,882	173	33	2,871,137	32.61	345,460	WAXR00000000	SRR10179113
MRSA70	504	3,594,008	542,695,208	189	31	2,869,703	32.61	333,066	WAXQ00000000	SRR10179112
MRSA700	497	3,013,346	455,015,246	158	31	2,881,570	32.70	289,415	WAUW00000000	SRR10179074
MRSA701	472	3,847,918	581,035,618	202	30	2,869,335	32.80	222,179	WAUV00000000	SRR10179073
MRSA702	439	3,806,952	574,849,752	198	51	2,897,062	32.70	289,645	WAUU00000000	SRR10179072
MRSA703	447	3,486,034	526,391,134	193	32	2,724,049	32.67	213,580	WAUT00000000	SRR10179070
MRSA704	460	3,636,896	549,171,296	192	24	2,861,835	32.74	486,123	WAUS00000000	SRR10179069
MRSA705	445	3,133,628	473,177,828	163	27	2,895,112	32.69	289,646	WAUR00000000	SRR10179068
MRSA707	449	3,522,546	531,904,446	185	30	2,868,800	32.61	304,506	WAUQ00000000	SRR10179067
MRSA708	448	3,180,238	480,215,938	170	38	2,823,372	32.74	191,364	WAUP00000000	SRR10179066
MRSA709	463	3,305,720	499,163,720	172	37	2,899,616	32.68	289,413	WAUO00000000	SRR10179065
MRSA71	527	4,365,444	659,182,044	230	28	2,869,327	32.61	305,136	WAXP00000000	SRR10179111
MRSA710	469	3,082,398	465,442,098	162	39	2,864,756	32.71	420,967	WAUN00000000	SRR10179064
MRSA711	496	4,104,888	619,838,088	213	30	2,907,254	32.63	345,464	WAUM00000000	SRR10179063
MRSA712	468	3,643,566	550,178,466	200	26	2,745,716	32.66	198,484	WAUL00000000	SRR10179062
MRSA713	495	3,418,280	516,160,280	175	69	2,945,485	32.70	289,646	WAUK00000000	SRR10179061
MRSA714	441	4,218,042	636,924,342	222	27	2,863,668	32.60	380,636	WAUJ00000000	SRR10179059
MRSA715	462	3,481,852	525,759,652	184	23	2,850,867	32.61	345,454	WAUI00000000	SRR10179058
MRSA716	471	4,091,464	617,811,064	212	31	2,911,450	32.63	283,748	WAUH00000000	SRR10179057
MRSA717	459	3,917,540	591,548,540	208	26	2,837,461	32.63	333,127	WAUG00000000	SRR10179056
MRSA718	534	4,225,508	638,051,708	222	28	2,871,939	32.61	284,113	WAUF00000000	SRR10179055
MRSA719	484	3,213,558	485,247,258	169	49	2,867,615	32.79	280,853	WAUE00000000	SRR10179054
MRSA72	467	3,986,734	601,996,834	209	50	2,874,486	32.75	205,960	WAXO00000000	SRR10179110
MRSA720	444	2,671,854	403,449,954	139	29	2,898,232	32.68	442,377	WAUD00000000	SRR10179053
MRSA721	655	4,241,198	640,420,898	228	27	2,808,909	32.61	241,918	WAUC00000000	SRR10179052
MRSA722	429	2,568,058	387,776,758	134	31	2,893,784	32.65	304,501	WAUB00000000	SRR10179051
MRSA723	480	3,033,028	457,987,228	157	32	2,909,093	32.64	282,763	WAUA00000000	SRR10179050
MRSA724	474	3,375,098	509,639,798	187	33	2,720,696	32.66	220,191	WATZ00000000	SRR10179048
MRSA725	540	3,348,842	505,675,142	175	36	2,892,888	32.62	243,923	WATY00000000	SRR10179047
MRSA726	455	2,505,498	378,330,198	139	25	2,716,855	32.67	219,751	WATX00000000	SRR10179046
MRSA727	474	3,710,920	560,348,920	193	27	2,904,858	32.63	375,095	WATW00000000	SRR10179045
MRSA728	461	3,755,872	567,136,672	195	36	2,912,730	32.64	333,120	WATV00000000	SRR10179044
MRSA729	428	3,406,082	514,318,382	182	29	2,823,905	32.60	299,344	WATU00000000	SRR10179043
MRSA73	507	4,534,946	684,776,846	241	41	2,846,312	32.71	215,893	WAXN00000000	SRR10179109
MRSA730	471	4,430,562	669,014,862	242	38	2,762,765	32.69	126,491	WATT00000000	SRR10179042
MRSA731	489	3,718,120	561,436,120	198	24	2,840,169	32.63	343,982	WATS00000000	SRR10179041
MRSA732	467	3,023,408	456,534,608	159	35	2,875,106	32.73	243,923	WATR00000000	SRR10179040
MRSA733	488	992,240	149,828,240	52	37	2,882,748	32.67	173,154	WATQ00000000	SRR10179039
MRSA734	449	3,321,522	501,549,822	176	46	2,854,666	32.70	161,449	WATP00000000	SRR10179037
MRSA735	500	2,878,300	434,623,300	152	33	2,850,136	32.71	173,749	WATO00000000	SRR10179036
MRSA737	546	3,750,316	566,297,716	197	30	2,867,880	32.61	343,582	WATN00000000	SRR10179035
MRSA738	515	3,253,946	491,345,846	176	38	2,798,198	32.70	156,320	WATM00000000	SRR10179034
MRSA739	504	4,533,356	684,536,756	237	63	2,882,875	32.70	222,067	WATL00000000	SRR10179033
MRSA74	539	4,253,572	642,289,372	219	82	2,929,845	32.72	66,876	WAXM00000000	SRR10179108
MRSA740	486	4,429,562	668,863,862	233	27	2,864,924	32.61	333,123	WATK00000000	SRR10179032
MRSA741	495	3,447,334	520,547,434	179	34	2,913,654	32.63	379,507	WATJ00000000	SRR10179031
MRSA742	465	4,040,380	610,097,380	213	33	2,868,322	32.61	283,987	WATI00000000	SRR10179030
MRSA743	444	4,053,710	612,110,210	222	44	2,762,637	32.62	106,067	WATH00000000	SRR10179029
MRSA744	507	4,670,204	705,200,804	242	31	2,909,579	32.63	300,478	WATG00000000	SRR10179028
MRSA745	454	3,066,886	463,099,786	160	30	2,896,923	32.69	289,415	WATF00000000	SRR10179026
MRSA746	465	2,861,980	432,158,980	158	29	2,738,985	32.73	244,156	WATE00000000	SRR10179025
MRSA749	461	2,881,656	435,130,056	151	40	2,890,590	32.66	197,752	WATD00000000	SRR10179024
MRSA75	490	4,630,334	699,180,434	242	27	2,883,659	32.66	333,122	WAXL00000000	SRR10179106
MRSA750	457	3,051,362	460,755,662	162	40	2,840,221	32.72	279,245	WATC00000000	SRR10179023
MRSA751	498	3,811,110	575,477,610	201	28	2,863,501	32.69	288,405	WATB00000000	SRR10179022
MRSA752	453	4,324,122	652,942,422	230	26	2,837,974	32.62	345,460	WATA00000000	SRR10179021
MRSA753	477	3,367,518	508,495,218	178	33	2,863,634	32.61	201,596	WASZ00000000	SRR10179020
MRSA754	431	4,180,350	631,232,850	217	199	2,909,153	32.68	47,630	WASY00000000	SRR10179019
MRSA76	404	3,201,396	483,410,796	169	29	2,866,225	32.62	345,460	WAXK00000000	SRR10179105
MRSA77	535	3,342,204	504,672,804	177	30	2,844,557	32.56	304,502	WAXJ00000000	SRR10179104
MRSA78	469	18,938,328	2,859,687,528	1,008	25	2,836,910	32.65	299,314	WAXI00000000	SRR10179103
MRSA79	505	4,755,926	718,144,826	259	32	2,770,266	32.70	200,135	WAXH00000000	SRR10179102
MRSA8	566	3,874,494	585,048,594	201	32	2,917,786	32.63	283,744	WAXG00000000	SRR10179101
MRSA80	490	4,449,382	671,856,682	231	31	2,913,262	32.63	345,459	WAXF00000000	SRR10179100
MRSA81	580	4,095,304	618,390,904	212	75	2,910,841	32.69	153,760	WAXE00000000	SRR10179315
MRSA83	546	4,524,148	683,146,348	240	34	2,844,125	32.64	304,681	WAXD00000000	SRR10179314
MRSA85	527	4,457,926	673,146,826	237	29	2,839,253	32.62	304,752	WAXC00000000	SRR10179313
MRSA86	550	3,954,150	597,076,650	213	41	2,804,242	32.76	199,663	WAXB00000000	SRR10179311
MRSA87	536	3,929,800	593,399,800	203	61	2,928,066	32.69	219,253	WAXA00000000	SRR10179310
MRSA88	544	3,411,670	515,162,170	188	27	2,742,253	32.68	234,118	WAWZ00000000	SRR10179309
MRSA89	511	4,417,348	667,019,548	242	20	2,755,367	32.67	247,479	WAWY00000000	SRR10179308
MRSA9	595	3,779,022	570,632,322	201	37	2,845,063	32.75	216,299	WAWX00000000	SRR10179307
MRSA90	409	2,959,362	446,863,662	159	39	2,818,569	32.71	387,589	WAWW00000000	SRR10179306
MRSA91	527	5,203,138	785,673,838	271	45	2,896,174	32.76	221,197	WAWV00000000	SRR10179305
MRSA92	570	2,741,320	413,939,320	142	31	2,923,613	32.62	304,505	WAWU00000000	SRR10179304
MRSA93	574	3,563,988	538,162,188	187	30	2,880,303	32.66	333,118	WAWT00000000	SRR10179303
MRSA94	512	4,462,482	673,834,782	231	35	2,922,102	32.62	377,842	WAWS00000000	SRR10179302
MRSA95	520	3,276,014	494,678,114	172	60	2,872,762	32.75	212,998	WAWR00000000	SRR10179300
MRSA96	445	3,755,668	567,105,868	194	43	2,917,478	32.64	191,715	WAWQ00000000	SRR10179299
MRSA97	405	3,122,276	471,463,676	173	27	2,731,544	32.70	234,329	WAWP00000000	SRR10179298
MRSA98	464	3,570,268	539,110,468	185	36	2,908,087	32.74	419,319	WAWO00000000	SRR10179297
MRSA99	484	3,843,378	580,350,078	203	26	2,863,314	32.64	307,780	WAWN00000000	SRR10179296
MRSAS284	460	6,213,650	938,261,150	328	30	2,856,374	32.62	333,124	WBAG00000000	SRR10179188
