# Erratum for Adediran et al., “Examination of 388 Staphylococcus aureus Isolates from Intensive Care Unit Patients”

**DOI:** 10.1128/mra.00708-22

**Published:** 2022-08-08

**Authors:** Timileyin Adediran, Stephanie Hitchcock, Lyndsay M. O’Hara, Jane M. Michalski, J. Kristie Johnson, David P. Calfee, Loren G. Miller, Tracy H. Hazen, David A. Rasko, Anthony D. Harris

**Affiliations:** a Institute for Genome Sciences, University of Maryland School of Medicine, Baltimore, Maryland, USA; b Department of Microbiology and Immunology, University of Maryland School of Medicine, Baltimore, Maryland, USA; c Department of Pathology, University of Maryland School of Medicine, Baltimore, Maryland, USA; d Division of Infectious Diseases, Weill Cornell Medicine, New York, New York, USA; e LA Biomedical Research Center at Harbor-UCLA Medical Center, Torrance, California, USA; f Department of Epidemiology and Public Health, University of Maryland School of Medicine, Baltimore, Maryland, USA

## ERRATUM

Volume 8, no. 46, e01246-19, 2019, https://doi.org/10.1128/MRA.01246-19. Page 1: This article was published on 14 November 2019 with Timileyin Adediran’s surname misspelled as “Adedrian” in the byline.

